# Case Report: severe pulmonary hypertension in a child with micronutrient deficiency

**DOI:** 10.3389/fped.2025.1478889

**Published:** 2025-01-31

**Authors:** Laure Pache-Wannaz, Cristiana Voicu, Laurence Boillat, Nicole Sekarski

**Affiliations:** ^1^ Pediatric Cardiology, Women-Mother-Child Department, Lausanne University Hospital and University of Lausanne, Lausanne, Switzerland; ^2^ Pediatric Intensive Care Unit, Women-Mother-Child Department, Lausanne University Hospital and University of Lausanne, Lausanne, Switzerland

**Keywords:** case report, pulmonary hypertension, thiamine deficiency, micronutrient deficiency, vitamin supplementation, selective food behavior

## Abstract

We describe the rare case of a previously healthy seven year-old boy, with an acute clinical onset of severe pulmonary hypertension. He recovered rapidly after vitamin supplementation. Patient history showed a highly selective food intake in the context of autistic features. Thiamine deficiency seemed to be the predominant causing factor aggravated by moderate iron deficiency and supposed vitamin C deficiency. Dietary impact on pulmonary pressures is still poorly understood, but it seems that micronutrient deficiency could be a rare cause of pulmonary hypertension. Relatively frequent in developing countries and mostly in infants, thiamine deficiency should not be forgotten as a potential etiology in the differential diagnosis when patient dietary history is particular.

## Introduction

Pulmonary hypertension in children is defined by a mean pulmonary artery pressure of >20 mmHg or a pulmonary vascular resistance index of ≥3 Wood units · m^2^ and has a wide differential diagnosis (1). The European Society of Cardiology recommends a highly complete work-up to find an aetiology and to treat these patients (2). In the case described, following these guidelines, most causes were excluded. In the specific context of the selective dietary behaviour of our patient, a micronutrient analysis was performed and showed multiple deficiencies. The effect of diet on pulmonary hypertension is still poorly understood, but it seems that micronutrient deficiency could be a rare cause of pulmonary hypertension (3) and should not be forgotten in the differential diagnosis.

## Patient informations and clinical findings

A seven year-old boy was referred to our center for acute heart failure. He complained of fatigue and exertion intolerance for a few months increasing in the last days. For 48 h, parents also noticed an altered general status, peripheral edema as well as a fast heartbeat. He had no infectious, respiratory or gastrointestinal symptoms.

The patient was in good somatic health with a growth that showed small stature: height 110 cm (<P3), weight 18 kg (P3), and BMI 14.9 kg/m2 (P25–50). The immunization schema was up to date and there was no significant past history except for autistic features with extremely selective behavior concerning food (only rice and yogurt intake since the age of 1.5-year-old). There was no familial history of congenital heart disease, sudden death, or genetic syndromes.

On physical examination, the patient was afebrile (36.5°C), mildly tachycardic (111 bpm) and hypertensive (116/88 mmHg). He was mildly tachypneic (respiratory rate 33/min, saturation 99% on room air). He presented with signs of mild dehydration (sunken eyes and dry mucosa) but with visible bilateral eyelid oedema. Cardiovascular examination revealed normal S1 and S2 sounds, with a 3/6 mesocardial nonradiating systolic murmur and a right ventricular heave on palpation. Sensitive hepatomegaly was noted with a liver edge 1.5 cm below the right costal margin. There was no splenomegaly. The child's extremities were cool, with a capillary refill time of three seconds, and he had bilateral pitting oedema of the ankles. The lungs were clear on auscultation. Despite hepatomegaly, the rest of abdominal status was normal. Neurological examination was difficult due to a partially cooperative patient in the context of autistic features, but no gross neurological deficiencies were noted. He was alert with good tone, no motor asymmetry of the face or the limbs and no nuchal rigidity.

## Diagnostic assessment, therapeutic intervention, follow-up and outcomes

**Figure 1:**
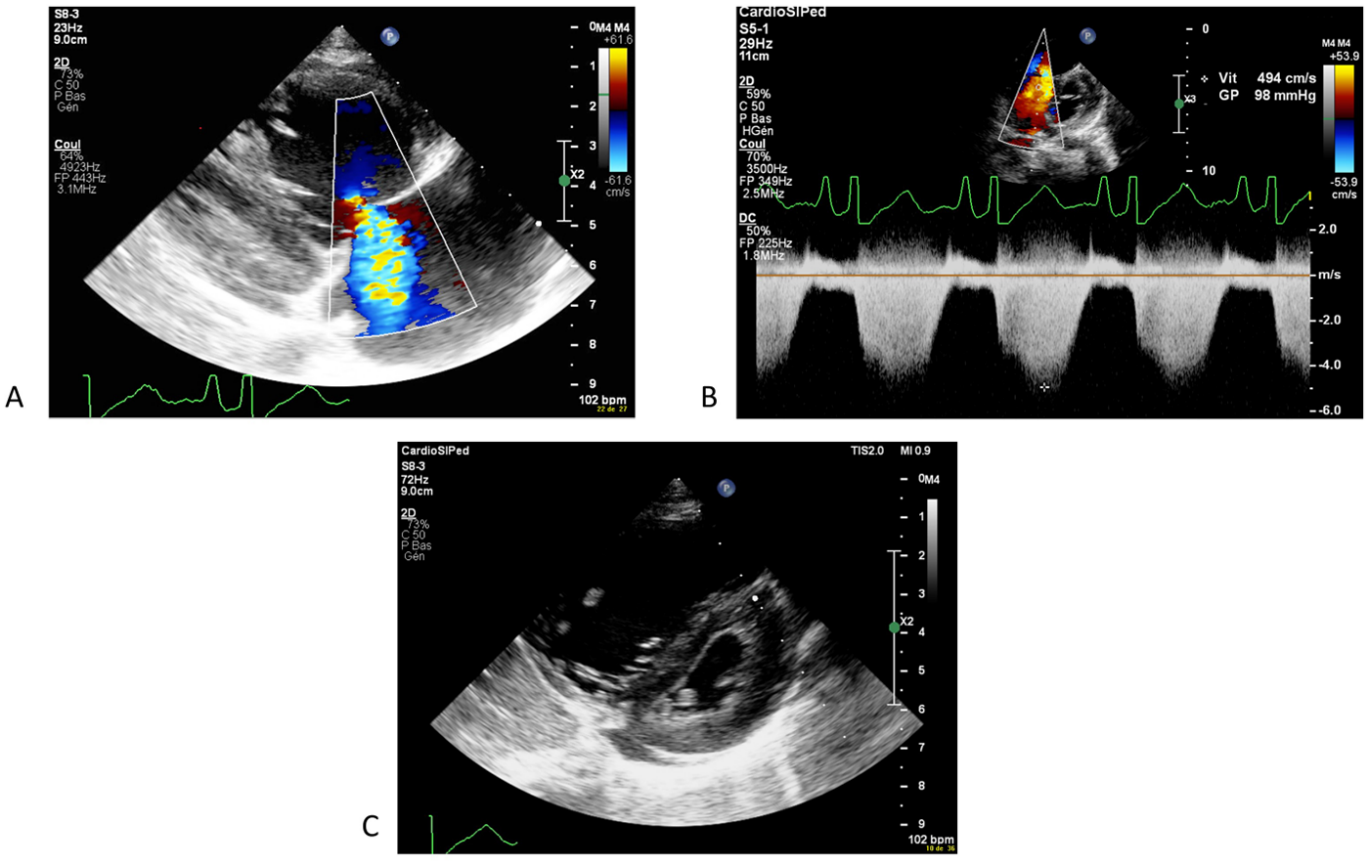
First echocardiography showing a severe pulmonary hypertension with tricupsid regurgitation **(A)** allowing a measure of right ventricular pressure of 98 mmHg on continous dopplrd **(B)** and a flattened interventricular septum **(C)**.

Initial investigations revealed cardiomegaly on x-ray (cardiothoracic index 0.62), an increased NT pro-BNP (14,973 ng/L) and troponin level (109 ng/L) on blood tests (Table 1). Electrocardiogram showed an image of a partial right bundle branch with rsR' in V1-V2 and a deep S wave in V6 for the patient's age (5 mV). Transthoracic echocardiography revealed normal cardiac anatomy but severe pulmonary hypertension with systolic right ventricular pressure of 98 mmHg (nearly isosystemic) based on severe tricuspid regurgitation and mean pulmonary arterial pressure of 53 mmHg based on moderate pulmonary regurgitation. The interventricular septum was flattened in systole (Figure 1). The right atrium, right ventricle, and pulmonary artery were dilated. Right ventricle function was impaired with a fractional area change of 22%, tricuspid annular plane systolic excursion of 0.85 cm (Z-score of −7.27), and signs of diastolic dysfunction. Left systolic ventricular function was preserved (left ventricular ejection fraction of 69%) but signs of diastolic dysfunction were present. There was a small amount of pericardial effusion. The patient was admitted to the pediatric intensive care unit and he was started on oxygen, milrinone, and diuretics. General support management in our pediatric intensive care unit relied also on micronutrient and vitamin supplementation for all the children, even without indicators of deficiency, due to the high metabolic stress in these very sick patients. These supplements consisted of intravenous thiamine (32.5 mg once daily), vitamin C (650 mg once daily), carnitine (200 mg four times a day), and multi vitamin (Supradyn Protovit®). Due to poor food intake, additional support with glucose and amino acid infusion was also started.

**Table 1:** Main laboratory values at hospitalization and at discharge.

	At hospitalization	10 days after discharge
Sodium (mmol/L)	135	138
Potassium (mmol/L)	4.5	4.0
Urea (mmol/L)	7.4	2.1
Creatinine (umol/L)	**62**	41
Albumin (g/L)	42	
Iron (umol/L)	**2.7**	
Ferritin (ug/L)	17	
NT-proBNP (ng/L)	**14,973**	<50
Troponin T (ng/L)	**109**	<13
Creatine kinase (U/L)	**237**	
Creatin kinase-MB (U/L)	24	
ASAT (U/L)	40	36
ALAT (U/L)	19	13
Alkaline phosphatases (U/L)	207	
Gamma-glutamyl transpeptidase (U/L)	26	
Total bilirubin (umol/L)	5	
Direct bilirubin (umol/L)	3	
Lactate deshydrogenase (U/L)	258	
Total homocysteine (umol/L)	7.5	
Thiamine (B1) (nmol/L)	**39.6**	**254** ^b^
Folate (B9) (nmol/L)	**8.5**	35.2
Cobalamine (B12) (pmol/L)	286	
Vitamin C (umol/L)	**<4** ^a^	

Abnormal values are in bold.

^a^ Vitamine C value could not be interpreted as sample was not preserved properly for a reliable analysis.

^b^ Values up to 270 nmol/L are expected with correct supplementation.

At additional investigations, chest CT scan showed no signs of parenchymal disease or pulmonary embolism; coagulation blood work was normal. Obstructive apnea was excluded as there was no evidence in the patient's history and none was observed during continuous monitoring in the intensive care unit. Laboratory findings showed no signs of liver disease and portal hypertension was excluded on abdominal ultrasound. No infectious causes were found. Thyroid values were normal. Immunologic and metabolic investigations showed no signs of vasculitis or metabolic disease. Micronutrient analysis was finally added to the laboratory work-up, as the context of extremely selective behavior concerning food intake could lead to suspicion of deficiency. These revealed an iron deficit of 2.7 umol/L (normal range 12.5–25.1 umol/L) with hemoglobin of 109 g/L, severe B1 deficit 39.6 nmol/L (normal range 78–143 nmol/L), and a folate of 8.5 nmol/L (normal range 11.3–47.6 nmol/L) (Table 1).

**Figure 2:**
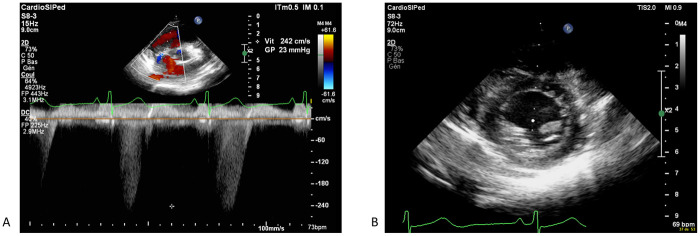
Echocardiography showing a significant improvement after 48 h of treatment, with regression of tricupsid regurgitation and complete normalizazion of right venticular pressure **(A)** with normal motion of the interventicular septum **(B)**.

**Figure 3:**
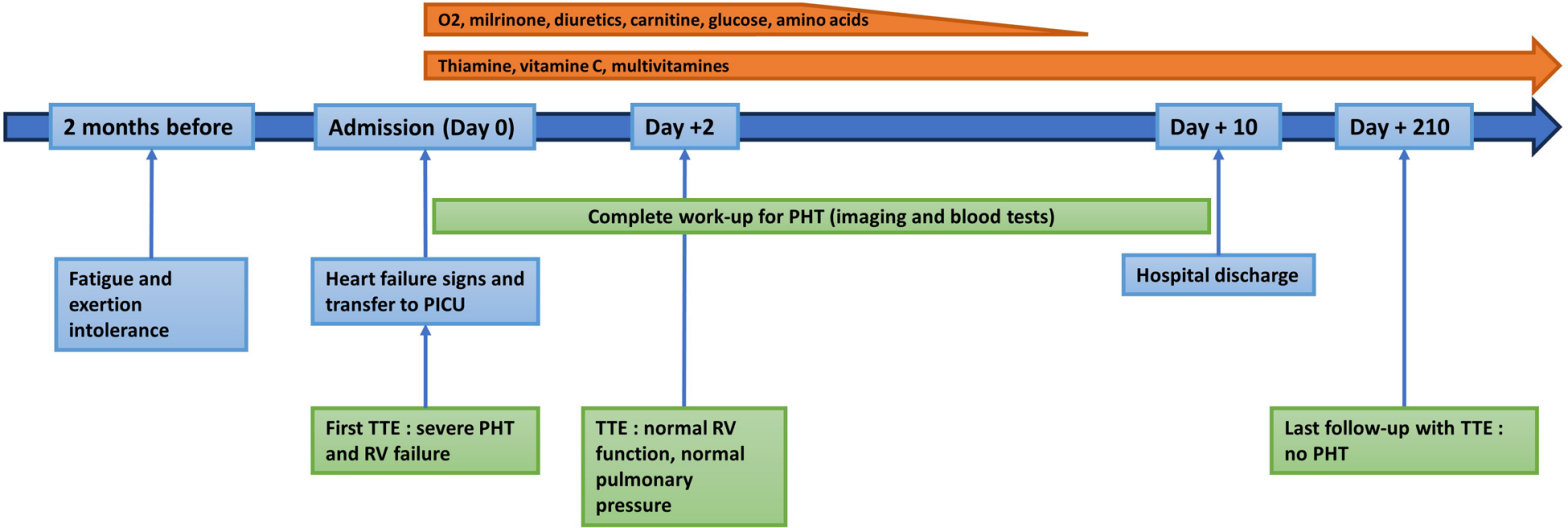
Timeline of the patient's history, the investigations, the treatments and the follow-up.

48 h after the onset of the aspecific pulmonary hypertension treatment and vitamin supplements, echocardiogram was repeated which showed significant improvement of right ventricular function (fractional area change of 40%, tricuspid annular plane systolic excursion of 19 mm (Z score - 0.27), regression of tricuspid regurgitation and almost complete normalization of right ventricular pressures of 23 mmHg (Figure 2). Oxygen, diuretics, and milrinone were progressively weaned within 3 days without increasing in pulmonary pressures. Cardiac catheterization was deemed to not be indicated. NT-proBNP result at seven days post-admission was 116 ng/L and the electrocardiogram showed the resolution of the abovementioned abnormalities. The patient was discharged from the hospital at day 10 post-admission, in good general condition, with a normal cardiovascular physical examination and an oral treatment of thiamine (100 mg once daily), vitamin C (100 mg once daily), iron-III hydroxide polymaltose (50 mg twice daily) and multivitamin solution at 20 drops per day (Supradyn Protovit®). All supplementations were given orally, mixed with yogurt, and accepted by the patient. A blood sample at 10 days after home discharge showed the resolution of B1 deficiency and no adverse effect of this supplementation occurs. Follow-up with echocardiogram assessment showed no relapse with at last follow-up at seven months after the episode (Figure 3).

## Discussion

Pulmonary hypertension is divided in five groups: pulmonary artery hypertension, pulmonary hypertension due to left-sided heart disease, pulmonary hypertension caused by lung disease or hypoxemia, chronic thromboembolic disease and pulmonary hypertension with unclear or multifactorial mechanisms (4). In this context, investigations should be broad to find an etiology that could be treated specifically, in addition to unspecific treatments of pulmonary vasculature dilatation. In our situation, all classical causes of pulmonary hypertension have been excluded and our patient could be classified in the group defined by pulmonary hypertension with unclear or multifactorial mechanisms.

The dietary impact on pulmonary hypertension is still poorly understood, however, some cases of micronutrient deficiency have been suggested as etiology in the literature. Additionally, patients with pulmonary hypertension are more susceptible to micronutrient deficiencies, as described in observational adult cohort (5). The most common association is between vitamin C, vitamin D, iron deficiencies and pulmonary hypertension. But many other micronutrient deficiencies were also observed, such as those of vitamin B12, vitamin K1 and selenium (6).

Vitamin C deficiency causing pulmonary hypertension in children is described in several case reports (7–11). It is the most described nutritional deficiency in the setting of pulmonary hypertension development. Pathogenesis is believed to be caused by low endothelial nitric oxide levels and an inappropriate activation of hypoxia-inducible transcription factors in ascorbic acid deficiency. Supplementation in vitamin C has the ability to fully and rapidly reverse the situation (12). Severe vitamin C deficiency (scurvy) is linked to other symptoms such as musculoskeletal involvement, mucosal bleeding, and gingivitis. In our case due to the wrong preservation of the blood sample tube, no reliable vitamin C dosage was available before the substitution began. Our patient had no clinical signs of scurvy, but a moderate vitamin C deficiency can not be totally excluded.

Vitamin D deficiency is suspected of having an impact on the development of pulmonary hypertension due to the immunomodulatory and anti-proliferative properties of this vitamin, specifically affecting endothelial cells and vascular smooth muscle cells. A correlation between pulmonary hypertension and low vitamin D levels was found in the adult population, as well as a link between the severity of deficiency and the severity of pulmonary pressures (13, 14). However, no evidence shows that it could directly cause pulmonary hypertension. It is rather supposed to be an aggravating factor in combination with other risk factors. In our case vitamin D was not assessed but our patient's diet does not put him at risk of deficiency in vitamin D.

Iron deficiency is also prevalent in patients with pulmonary hypertension. This condition impacts exercise capacity and NYHA functional class in adults, regardless of whether anemia is present (15, 16). Iron deficiency creates a constriction of the pulmonary circulation but some authors suspect that it may be a consequence of the condition, rather than just a causing factor. Based on the molecular mechanisms, these patients appear to have dysregulated iron homeostasis, primarily due to high hepcidin levels. Hepcidin plays a role in the remodeling of pulmonary circulation by promoting the proliferation of smooth muscle cells (17). Whether oral iron supplementation is effective in this condition remains unresolved, as high hepcidin levels trigger iron malabsorption. Our patient had a very low iron level with mild anemia, but his ferritin was in the low normal range. We chose to try an iron oral substitution that was prescribed for him at hospital discharge.

Thiamine deficiency, also called Beriberi, is nowadays mostly described in low socio-economic setting of developing countries, where the diet is mostly based on rice, with poor access to high micronutrient containing food. The human body relies only on exogenous sources of thiamine, as no endogenous synthesis is done. Daily intake recommended for children is 0.2 mg/day during early infancy and then steadily increases with age to adult levels (1.1–1.2 mg/day) (18). The main source of thiamine is found in cereals, whole grains, pork, salmon, and some peas and vegetables. Yogurt contains also small quantity of thiamine, but not enough to fulfill daily requirements. Other causes of thiamine deficit can be alcoholism, malignancy, burns, loop diuretics use, dialysis, and use of parenteral nutrition without micronutrient supplementation (19, 20). Two clinical presentations of Beriberi coexist. The first is described as the dry form, consisting mainly of neurological impairment and the second is described as the wet form, represented by a predominent cardiovascular symptomatology leading to a congestive heart failure but not often pulmonary hypertension (21). Most described cases of pulmonary hypertension due to thiamine deficiency happened in infants, who are at higher risk due to very low levels of thiamine in the breast milk of deficient mothers in developing countries (22). Older children, as well as adults, tend to present with more encephalopathic and neuropathic forms, but some adult cases of pulmonary hypertension have been reported (20, 23, 24). In our case, no gross neurologic features were found. This is most likely due to individual variability in Beriberi presentation. However, it should also be emphasized that the patient had a limited expressive capacity and did not fully collaborate during physical examination, due to his autistic features and some minor neurologic anomalies could not have been detected.

Classic cardiovascular effects of thiamine deficiency are multiple and the specific pathway leading to pulmonary hypertension is not well understood. First, thiamine deficit causes low systemic vascular resistance due to peripheral vasodilatation, as well as capillary leak. This induces a high cardiac output state, where cardiac output increases disproportionately. Two supposed mechanisms for pulmonary hypertension in this situation have been advocated (25): the first one relies on superoxide anions, reactive oxygen, and nitrogen species creation due to thiamine deficiency. Shear stress on pulmonary endothelium created by high cardiac output elevated, even more, these substances (26). They cause direct vasoconstriction and also impact nitric oxide, thereby inducing further constriction. The other mechanism implies an energy depletion due to mitochondrial failure, as thiamine acts as a cofactor for energy production. Cardiomyocytes are damaged and cardiac failure happens with, among other things, an elevation in left ventricular end-diastolic pressure that creates pulmonary venous hypertension. It is noticed that despite these hypotheses, most Beriberi cases describe a normokinetic or hyperkinetic left ventricle (27), as in our patient.

Our center already published in 2020 another case of thiamine deficiency with cardiovascular compromise and persistent lactate acidosis (28). Due to its action of cofactor for aerobic glycolysis, lack of thiamine leads to lactate accumulation through anaerobic glycolysis. This situation is reversible rapidly, as soon as supplementation is started. In that case however, no pulmonary hypertension was noted.

No evidence regarding the dosage for thiamine supplementation in children is available, nor its duration. In most studies, authors use an initial dose of 100–150 mg intravenous to reverse pulmonary hypertension for about 3 days, then a lower substitutive dose is given during some months. The administration of thiamine shows remarkable effect, and no adverse effects were found in all analyzed cases. An Indian study with 250 only breast-feed infants who presented with severe pulmonary hypertension without any other heart or lung disease were treated with 100 mg intravenous thiamine. Supplementation resolved the situation completely in 92% of patients with clinical improvement as early as 6–24 h and echocardiographic improvement at 24–48 h (26). Another study shows reversed biochemical abnormalities due to thiamine deficiency within 2–4 h (25).

Combined micronutrient deficiencies could also be found in patients with pulmonary hypertension, and it is not easy to detect which deficiency is the leading cause and/or if a sum of small amplitude deficiency is sufficient to trigger this clinical picture. In most cases, it happens in patients with selective behavior concerning food, as in our case. Another case report from Boston Children's Hospital (29) described a child with pulmonary hypertension and musculoskeletal complaints in the context of multi-micronutrient deficit (vitamin C, thiamine, B6, B12, and vitamin D). As well as in our patient, the child has autistic features with a very high selectivity concerning food. In this setting, clear symptoms of scurvy were present, and it was the first diagnosis.

In conclusion, we present an unusual case of severe pediatric pulmonary hypertension with right heart failure secondary to multimicronutrient deficiency, which resolved rapidly with vitamin and mineral repletion. Although there are multiple case reports of pulmonary hypertension associated with micronutrient deficiencies, to our knowledge this is the first to report on severe combined deficiency. This case highlights the importance of a thorough history and thorough investigation to look at many micronutrients, including less commonly reported deficiencies such as thiamine. Prompt recognition of micronutrient deficiencies is crucial to allow repletion and prevent the progression of disease.

## Patient perspective

The patient's perspective does not seem justified in our case report, as the patient cannot express himself properly because of his autistic features.

## Data Availability

The datasets presented in this article are not readily available because of ethical and privacy restrictions. Requests to access the datasets should be directed to the corresponding author.
